# Delineating *Anaplasma phagocytophilum* Ecotypes in Coexisting, Discrete Enzootic Cycles

**DOI:** 10.3201/eid1512.090178

**Published:** 2009-12

**Authors:** Kevin J. Bown, Xavier Lambin, Nicholas H. Ogden, Michael Begon, Gill Telford, Zerai Woldehiwet, Richard J. Birtles

**Affiliations:** University of Liverpool, Liverpool, UK (K.J. Bown, M. Begon, Z. Woldehiwet, R.J. Birtles); University of Aberdeen, Aberdeen, UK (X. Lambin, G. Telford); Public Health Agency of Canada, Saint-Hyacinthe, Quebec, Canada (N.H. Ogden)

**Keywords:** Bacteria, anaplasma, vector-borne infections, ticks, arthropod vectors, infectious disease reservoirs, communicable disease transmission, biological adaptation, research

## Abstract

The emerging tick-borne pathogen *Anaplasma phagocytophilum* is under increasing scrutiny for the existence of subpopulations that are adapted to different natural cycles. Here, we characterized the diversity of *A. phagocytophilum* genotypes circulating in a natural system that includes multiple hosts and at least 2 tick species, *Ixodes ricinus* and the small mammal specialist *I. trianguliceps*. We encountered numerous genotypes, but only 1 in rodents, with the remainder limited to deer and host-seeking *I. ricinus* ticks. The absence of the rodent-associated genotype from host-seeking *I. ricinus* ticks was notable because we demonstrated that rodents fed a large proportion of the *I. ricinus* larval population and that these larvae were abundant when infections caused by the rodent-associated genotype were prevalent. These observations are consistent with the conclusion that genotypically distinct subpopulations of *A. phagocytophilum* are restricted to coexisting but separate enzootic cycles and suggest that this restriction may result from specific vector compatibility.

## Abstract 2

Genetically distinct subpopulations have adapted to different niches

The tick-transmitted bacterium *Anaplasma phagocytophilum* is the causative agent of granulocytic anaplasmosis, an infection of medical and veterinary importance that is widely encountered across the temperate zones of the Northern Hemisphere (*1*–*3*). Although considerable effort has been put into determining the natural diversity of *A. phagocytophilum* (*4*–*6*), our understanding of ecologic and evolutionary processes that underlie this diversity remains far from complete. *A. phagocytophilum* has been detected in the blood of a wide range of mammals and in several different *Ixodes* species, which suggests that it is a generalist parasite with the capacity to exploit multiple hosts and vectors (*1*–*3*,*5*–*9*). However, evidence for the existence of subpopulations that are adapted to specific reservoir host species has recently been forthcoming (*7*,*9*,*10*), and these subpopulations appear to possess differing potential to be a public health threat (*7*,*9*,*10*). This phenomenon has also been described within another tick-borne generalist species–complex, *Borrelia burgdorferi* sensu lato (*11*) and, more recently, within the 1 generalist member of this complex, *B. burgdorferi* sensu strictu (*12*,*13*). As yet, no evidence has shown that subpopulations of either *A. phagocytophilum* or *B. burgdorferi* have adapted to different *Ixodes* species as vectors.

Knowledge of the existence of host- or vector-adapted subpopulations is important given the public health consequences of multivector transmission by these pathogens. For example, we and other researchers (*14*–*19*) have hypothesized that pathogen populations maintained in efficient tick-rodent cycles by nidicolous (nest-living and host-specialist) ticks, such as *I. trianguliceps* in the United Kingdom and *I. spinipalpis* and *I. minor* in the United States, could spill over into the human population due to the co-occurrence of sympatric exophilic (and human-biting) tick species such as *I. ricinus* in the United Kingdom and *I. pacificus* and *I. scapularis* in the United States.

The purpose of this study was to characterize the diversity of *A. phagocytophilum* strains circulating in a natural multihost, multivector system and to determine whether the observed diversity had any ecologic basis. We obtained compelling evidence to support the proposition that different subpopulations of *A. phagocytophilum* exploit different tick and mammal species and, as a result, occur in discrete enzootic cycles even though both vectors and hosts are sympatric.

## Materials and Methods

### Natural Multihost, Multivector Study System

Kielder Forest is a managed plantation forest dominated by Sitka and Norway spruce that covers 620 km^2^ in northern England (55°13′N, 2°33′W). The harvesting of timber generates clearcut areas of 5–12 ha, which are subsequently replanted and progress through grassland and the thicket stage after 12–15 years. The most abundant mammal species encountered within clearcut areas is the field vole (*Microtus agrestis*), which exhibits multiannual population cycles in which densities can reach >700/ha (*20*). Roe deer (*Capreolus capreolus*) are also abundant at an estimated density of 3 deer/km^2^ across the forest and are frequent visitors to clearcut areas (*21*). The presence of *I. ricinus* and *I. trianguliceps* ticks in clearcut areas has been documented (*18*,*19*).

### Monitoring of Host and Vector Populations

Protocols for the handling and sampling of wild rodents were approved by the University of Liverpool Committee on Research Ethics and were conducted in compliance with the terms and conditions of licenses awarded under the UK Government Animals (Scientific Procedures) Act, 1986. Voles were trapped at 4-week intervals from March 2004 through November 2005 (excepting December 2004 and February 2005) at 4 principal study sites that were 3.5 km–12 km apart. Each animal captured was processed as described previously and a blood sample was taken from the tail tip (*19*). Voles were examined for ticks, with all larvae being removed and stored in 70% ethanol for identification (*22*,*23*) before releasing the animal at the point of capture. Nymph and adult ticks were not removed to minimize any effect on the transmission of tick-borne infections, which were being studied as part of an extensive longitudinal program. Host-seeking *I. ricinus* nymphs and adults were collected at monthly intervals from the principal study sites from March 2004 through September 2005 as previously described (*19*) and from 17 additional sites widely distributed across the Kielder Forest District. Collected ticks were stored and identified as described above. Roe deer blood samples were collected from January 2004 through July 2006 from animals culled throughout the forest and stored in EDTA-containing tubes at –20°C.

### Host Bloodmeal Source Identification

The relative importance of different species as hosts for *I. ricinus* larvae was determined as previously described (*24*). Probes for the following taxa were used: *Myodes* spp., *Apodemus* spp., *Microtus agrestis*, *Sciurus* spp., *Sorex araneus*, *Meles meles*, and *C. capreolus,* together with a generic “bird” probe (*24*).

### Monitoring of *A. phagocytophilum* Genotypes

Crude nucleic acid extracts were prepared from blood samples and host-seeking *I. ricinus* nymphs as previously described (*11*). The presence of *A. phagocytophilum* DNA in each extract was assessed by a real-time PCR (*25*).

Genotyping of *A. phagocytophilum* strains exploited sequence variation at 3 genetic loci, 16S rDNA, *msp4,* and DOV1. Fragments of *msp4* and 16SrDNA were amplified and analyzed as described (*18*,*25*). DOV1 is a noncoding region of ≈275 bp lying immediately downstream of a previously described variable number tandem repeat (VNTR) locus (*6*). Amplification of this locus was achieved by using seminested PCR. The first round of amplification contained 10 ρmol of each of the primers DOV1f (5′-GAT CTA TGA ATT GCY RGT GTT ATA-3′) and DOV1r1 (5′-ACA TTG TCA ATT TCT CAC CAT-3′), 12.5 µL of 2× Master Mix (Abgene, Epsom, UK), 1 µL of nucleic acid extract and sterile H_2_O to a final volume of 25 µL, which was subjected to a thermal program of 95°C for 3 min, then 35 cycles of 95°C for 10 s, 58°C for 10 s, and 72°C for 50 s, then a final extension step of 72°C for 5 min. The second round of amplification incorporated 1 µL of first-round product into a reaction containing 10 ρmol of each of the primers DOV1f and DOV1r2 (5′-CAA YRC ACR YAC ATT TCT AGG A-3′), 22.5 µL of Reddymix (ABgene), made up to a final volume of a 25 µL with sterile H_2_O. This reaction mix was subjected to the same thermal program as used for the first round of amplification. DOV1 amplicons were sequenced directly by using the second round primers. DNA sequences from all 3 loci studied were verified, assembled and aligned by using Chromas Pro (Technelysium Pty Ltd, Tewantin, Queensland, Australia) and MEGA4 software (*26*). Sequence similarity calculations and phylogenetic inferences were conducted by using MEGA4 software (*26*).

## Results

### Monitoring of Host and Vector Populations

**Figure 1:**
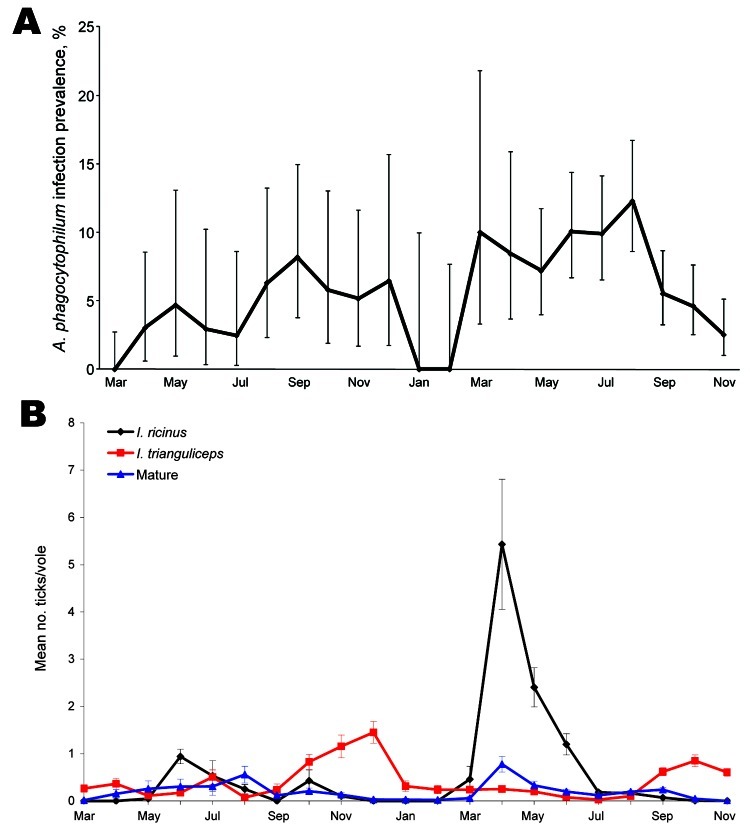
Prevalence of *Anaplasma phagocytophilum* infection in field voles (A) and of infestation of *Ixodes ricinus* tick larvae (black line), *I. trianguliceps* tick larvae (red line), and *I. ricinus*/*I. trianguliceps* adult females and nymphs (blue line) on field voles (B) during March 2004–November 2005. Error bars represent exact binomial 95% confidence intervals (A) or SEM (B).

A total of 2,926 blood samples from 1,503 voles at the 4 study sites was obtained. Similar numbers of voles were encountered at each site and the population size at all sites fluctuated in a broadly synchronous manner, in keeping with the well-documented seasonal and multiannual population cycles (*27*). *A. phagocytophilum* DNA was detected in 183 (6.3%) of the blood samples, representing 157 (10.4%) of individual animals tested. Except for the bacterium being seemingly absent from 1 site in 2004, the seasonal variation in prevalence of infection was similar at all sites, with infections disappearing over winter, before reappearing in the spring and persisting until late autumn (Figure 1, panel A).

Of the 3,378 ticks that were recorded on the surveyed voles, 83.6% (2,823) were larvae, 13.4% (454) were nymphs, and 2.9% (101) adults. Approximately equal numbers of *I. ricinus* (1,618, 57.3%) and *I. trianguliceps* (1,205, 42.7%) were identified among the larvae, the seasonal dynamics of which are shown in Figure 1, panel B. *I. ricinus* larvae were most abundant in late spring/early summer, whereas *I. trianguliceps* larvae were most abundant in late autumn. The dramatic spike in the number of *I. ricinus* larvae recorded in May 2005 resulted from a small number of voles at one of our principal study sites having an extremely high number of larvae. Although nymph and adult ticks were not removed from voles (so could not be identified to species), their numbers were recorded. Of relevance to this study, virtually no nymphs or adults were observed on voles between November and April (Figure 1, panel B). The absence of the life stages that are capable transmitters of *A. phagocytophilum* underlies the disappearance of infections in voles during winter.

**Figure 2:**
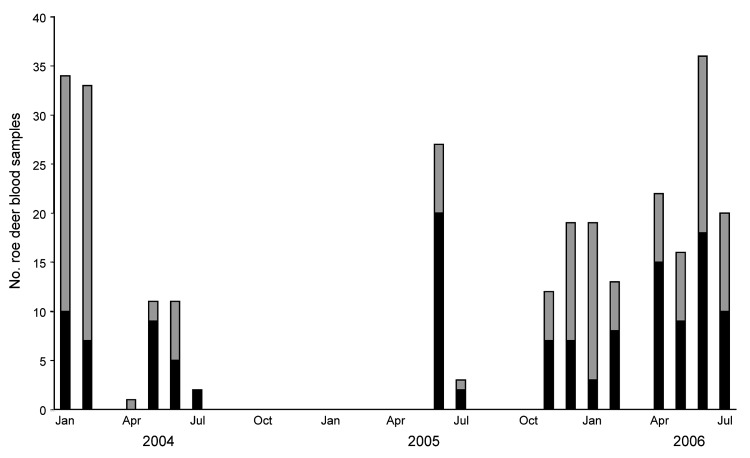
Number of *Anaplasma phagocytophilum*–infected (black bars) and uninfected (gray bars) animals encountered among Kielder Forest District roe deer sampled during January 2004–July 2006.

Blood samples were collected from 279 roe deer and *A. phagocytophilum* DNA was detected in 132 (47.3%) of these samples. Infections were detected throughout the year, with infection prevalence consistently high during the late spring/early summer of the years surveyed (Figure 2).

In total, 4,984 nymphs, 680 adult males, and 656 adult female host-seeking *I. ricinus* ticks were collected by dragging. The seasonal dynamics of both life stages have been presented elsewhere (*19*). *A. phagocytophilum* DNA was detected in 30 of 4,256 nymphs tested (0.7%), 9 of 263 adult females (3.4%) and 8 of 321 adult males (2.5%). Infected nymphs were encountered at 10 different sites. Infected host-seeking nymphs were collected during the same dragging session on only 8 occasions, suggesting that, for the most part, infected nymphs had fed on different animals.

### Host Bloodmeal Source Identification

Bloodmeal source identification was attempted on 399 host-seeking *I. ricinus* nymphs and unambiguous results were obtained for 105 ticks (26.3%). These ticks were obtained from dragging sessions throughout 2004 (87 ticks) and 2005 (18 ticks) from the 4 principal study sites. Sixty-two (59.0%) showed evidence of having fed on voles as larvae, 18 (17.1%) fed on birds, 15 (14.3%) fed on deer, and the remaining 10 (9.5%) fed on small mammal species other than field voles.

### Monitoring of *A. phagocytophilum* Genotypes

Comparison of partial 16S rDNA sequences obtained from 5 infected voles and 5 infected deer showed 4 highly similar (>99%) sequence types. All voles were infected with a sequence type that was identical to one previously associated with various ruminant species (e.g., Old Sourhope, GenBank AY176591). Three 16S rDNA sequence types were obtained from the deer samples, 2 of which had been previously reported associated with a variety of animals and tick species across the Northern Hemisphere (e.g., GenBank AF481850 and AJ242783), but the third sequence type was new. Although comparison of 16S rDNA sequence types was useful in confirming that detected DNAs were derived from strains of *A. phagocytophilum*, the insensitivity of this locus for intraspecies delineation led us to attempt sequence typing on the basis of a more variable locus, *msp4* (*6*,*28*).

Unambiguous sequence data were obtained from amplicons derived from 45 infected roe deer, 48 infected voles, and 21 infected host-seeking *I. ricinus* nymphs and adults. For each host or vector, the samples came from across the range of sites, seasons and years of study. Seven different *msp4* sequence types were obtained from infected roe deer. One sequence type was detected in most (30) samples. This and a second sequence type had previously been encountered among European deer, while the 5 remaining sequence types were new. Four *msp4* sequence types were encountered among the infected host-seeking *I. ricinus* ticks, all of which were also detected in roe deer. The most commonly encountered sequence type, which infected 17 ticks, was the same as that found most frequently among infected deer. The partial *msp4* sequences obtained from 48 infected field voles were all indistinguishable from one another.

**Figure 3:**
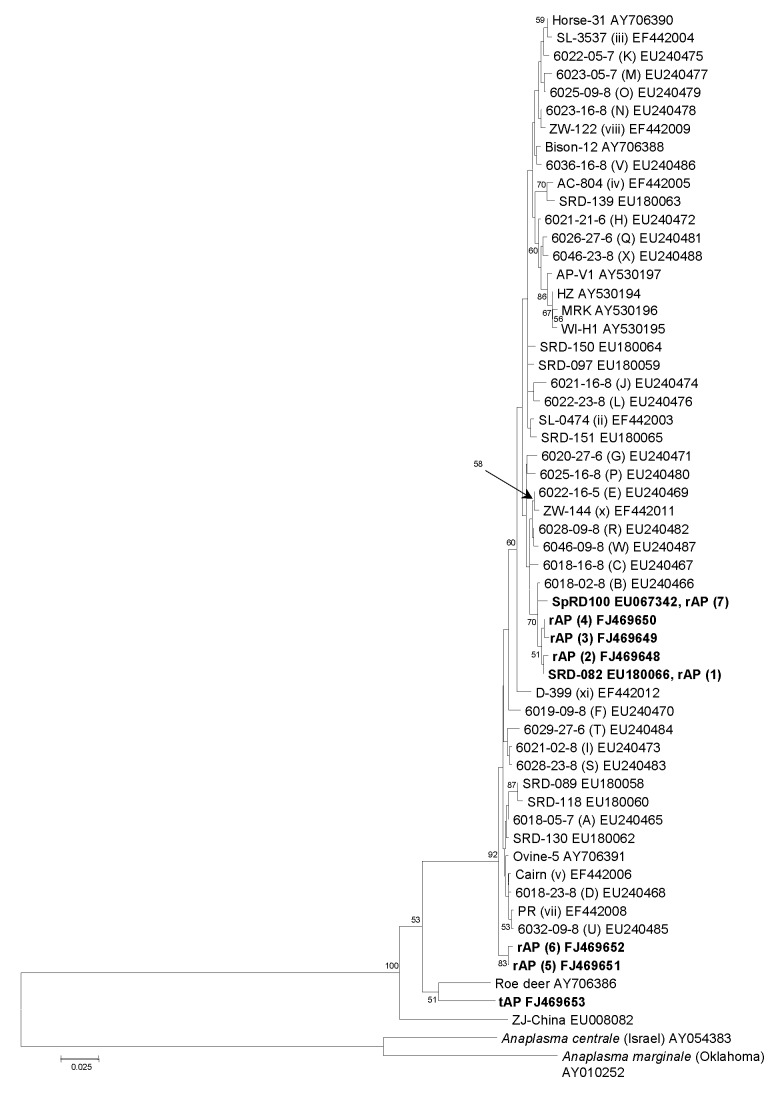
Phylogenetic tree inferred from alignment of *Anaplasma phagocytophilum msp4* sequences obtained in this study or available from GenBank. Inference was made by using the neighbor-joining algorithm. The stability of proposed branching order was assessed by bootstrapping (1,000 replicates). At nodes present in >50% of replicates, the percentage of replicates possessing the node is indicated. The GenBank accession numbers of the new *msp4* sequences obtained during this study (in **boldface**) are included in the strain designations. rAP sequence types were detected in questing *Ixodes ricinus* ticks and roe deer, and the tAP sequence type was detected in voles. Scale bar indicates nucleotide substitutions per site.

Phylogenetic analysis, based on an alignment of the 50 *A. phagocytophilum msp4* sequence types present in GenBank (as of August 1, 2008), the 6 new alleles reported in this study, and homologous sequences available for the closely related species *A. marginale* and *A. centrale*, was used to infer the relative evolutionary positions of the *A. phagocytophilum* strains encountered in this study. The 5 new sequence types obtained from roe deer and host-seeking *I. ricinus* ticks lay within a cluster of closely related sequence types that also included the 2 other sequence types recovered from roe deer and *I. ricinus* ticks that had been previously encountered elsewhere (Figure 3). This well-supported cluster comprised 50 of the 53 *A. phagocytophilum msp4* sequence types reported to date and was characterized by short intersequence type evolutionary distances and included strains associated with wild and domesticated ruminants, companion animals, and humans.

Three *A. phagocytophilum* sequence types lay outside this cluster (Figure 3) and included types specifically associated with voles in this study, one associated with Chinese rodents (ZJ-China) (*8*), and one obtained from an infected roe deer in Germany (“roe deer”) (*5*). The evolutionary distances between these 3 sequence types were markedly longer than those between the sequence types within the large cluster, and although maximum parsimony analysis indicated a shared line of descent for the vole-associated and roe deer–associated sequence types, this branching order was not strongly supported when distance matrix– or minimum evolution–based methods of inference were used, and no approach suggested a clustering of either of these sequence types with ZJ-China.

**Figure 4:**
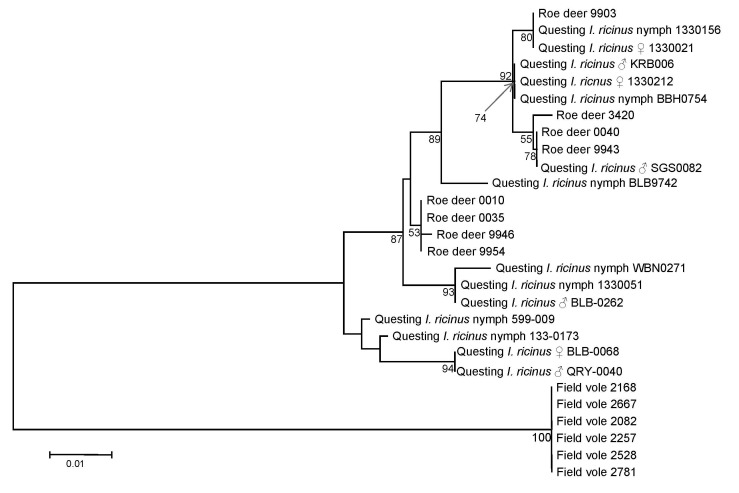
Phylogenetic tree inferred from alignment of *Anaplasma phagocytophilum* DOV1 sequence types obtained in this study. Inference was made by using the neighbor-joining algorithm. The stability of proposed branching order was assessed by bootstrapping (1,000 replicates). At nodes present in >50% of replicates, the percentage of replicates possessing the node is indicated. The DOV1 sequences obtained in this study have been deposited in GenBank under the accession nos. FJ469653–FJ469666. Scale bar indicates nucleotide substitutions per site.

Examination of DOV1 sequences supported the *msp4*-based analysis. Unambiguous DOV1 sequences were obtained from DNA extracts derived from 8 infected deer, 6 infected field voles, and 14 host-seeking *I. ricinus* ticks. A total of 13 different DOV1 sequence types were obtained; all infected voles yielded the same sequence type, whereas infected deer yielded 5 different sequence types, and infected host-seeking ticks yielded 9 different sequence types. Two sequence types were associated with both deer- and host-seeking *I. ricinus* ticks. Phylogenetic analysis inferred that DOV1 sequence types associated with deer and host-seeking *I. ricinus* ticks were closely related to one another, whereas sequence types associated with voles had markedly diverged (Figure 4). This phylogeny is congruent with that derived from *msp4* data.

## Discussion

Although considerable effort has been put into exploring the genetic diversity of *A. phagocytophilum* (*4*–*6*), the public and veterinary health value of this endeavor has been limited by the failure to identify an ecologic basis for the genotypic variation encountered. Recent studies in the United States have begun to resolve this problem, with the identification of apparent host-preference ecotypes among different 16S rDNA sequence types of *A. phagocytophilum* (*7*,*9*,*10*). One particular sequence type, referred to as Ap-variant 1, appears to exploit only white tailed deer (*Odocoileus virginianus*) as a reservoir host, whereas other variants, including that associated with human granulocytic anaplasmosis (Ap-ha), exploit white-footed mice (*Peromyscus leucopus*), a species that has long been implicated as a important reservoir for *A. phagocytophilum* in North America (*29*). In Europe, the epidemiology of *A. phagocytophilum* infections appears to be quite different from that in the United States. Although far fewer human cases have been reported, infections in livestock are common and represent a large financial burden to the industry (*30*). Surveys of strains infecting European livestock and wild-living ungulates have found numerous 16S rDNA sequence types, including Ap-ha and Ap-variant 1 (*6*,*31*), indicating that this locus is not a marker of the same *A. phagocytophilum* ecotypes present in the USA. In this study we have begun to unravel the ecologic significance of the genetic diversity present among European *A. phagocytophilum* strains by genotyping strains circulating in a natural multi-host, multi-vector system and correlating the genotypes we encountered with the provenance of the characterized strains. Our efforts have resulted in the discovery that field voles serve as a reservoir host for a unique genotype of the species that has markedly diverged from those genotypes encountered in wild roe deer and host-seeking *I. ricinus* nymphs and adults. This discovery is incompatible with the hypothesis that voles, *I. ricinus* ticks, and roe deer are all part of the same enzootic cycle but instead provides compelling evidence for at least two coexisting yet distinct enzootic cycles, one involving roe deer as hosts and *I. ricinus* ticks as vectors and another with field voles as hosts. As we have previously reported, *A. phagocytophilum* can be maintained in the absence of *I. ricinus* ticks in a natural cycle involving small mammals and *I. trianguliceps* ticks (*32*), and that, even when present in abundance, *I. ricinus* ticks do not play a major role in this cycle (*19*). *I. trianguliceps* ticks, which occur abundantly in our study system, are almost certainly a component of the enzootic cycle that includes field voles.

The results of our study are not compatible with the hypothesis that pathogen populations maintained in an enzootic rodent–nidicolous tick cycle could spill over into humans or livestock because of the co-occurrence of sympatric exophilic tick species (*14*–*19*). Through the use of host bloodmeal source identification, we demonstrated that *I. ricinus* larvae had ample opportunity to acquire *A. phagocytophilum* infection from voles (over half the questing *I. ricinus* nymphs we tested fed on voles as larvae). However, we found no evidence of the vole-associated genotype in host-seeking *I. ricinus* nymphs. This result suggests that, *I. ricinus* larvae are, at best, inefficient vectors of the vole-associated *A. phagocytophilum* genotype, thereby ostensibly removing the potential “bridge” out of the enzootic cycle that includes voles and *I. trianguliceps*. Notably, we did not detect deer/*I. ricinus* tick–associated *A. phagocytophilum* genotypes in voles despite previously observing *I. ricinus* nymphs feeding on these hosts (*25*). Because we did not remove nymph or adult ticks infesting our surveyed rodents in this study, we were unable to distinguish between *I. ricinus* and *I. trianguliceps* ticks, so were unable to gauge the frequency with which the former were encountered, although we believe that most nymphs on field voles are *I. trianguliceps* (*18*). Thus, this absence may result either from voles not being susceptible to deer/*I. ricinus*–associated genotypes or simply because encounters between infected *I. ricinus* nymphs and voles occur only rarely.

Although vector specialization by arthropod-transmitted pathogens is common (*33*), many of those that are tick-transmitted exploit more than 1 species (*15*–*19*).Also, clear evidence exists for local adaptation, whereby pathogens exhibit greater infectivity in local vector populations than those that are geographically distinct (*34*,*35*), although this phenomenon was not encountered for *A. phagocytophilum* (*36*). We report evidence for the adaptation of different genotypes of the same pathogen species to transmission by different but coexisting vector species. We are planning laboratory transmission studies to determine the extent to which this adaptation represents complete specialization of genotype to vector. Nonetheless, the data we have already obtained from our field studies provide a clear insight into the ecologic consequences of this adaptation; in other words, of what is, rather than what may be, happening. *A. phagocytophilum* has a wide geographic distribution, and numerous members of the *Ixodes* genus have been implicated in its transmission. Thus, plenty of scope remains for further exploration of vector specificity by subpopulations of the pathogen. The transmission of *A. phagocytophilum* in the laboratory has been reported (*29*) and, subsequently, efforts have been made to examine interstrain variation in the dynamics of this process (*37*). These include a demonstration that strains from the western United States that are naturally transmitted by *I. pacificus* ticks can be transmitted by *I. scapularis* ticks in the laboratory (*35*), which suggests that not all *A. phagocytophilum* strains have adapted to exploit only a single vector species. In addition, *I. scapularis* serves as a vector for both Ap-ha and Ap-variant 1, the 2 *A. phagocytophilum* genotypes that possess different host specificities (*7*,*9*,*10*). These observations, taken together with those made in the current study, provoke the conclusion that although the species as a whole can be considered a generalist, *A. phagocytophilum* embraces a consortium of distinct ecotypes that have evolved a range of strategies to facilitate their own perpetuation. Whether host or vector specialization is the more commonly adopted strategy remains to be explored.

From an infection control perspective, it is important to recognize that cryptic transmission cycles of tick-borne pathogens maintained by nidicolous ticks have substantial human and veterinary health risk implications when sympatric exophilic tick vectors act as a bridge to potentiate human or livestock infections. However, our study shows the value of a more profound understanding of the diversity of the transmission cycles and pathogens on which to base estimation of the environmental health hazard: discrete coexisting transmission cycles can be associated with dilution of the abundance of tick-borne pathogens when, at first sight, an augmentation would be the expected outcome.
